# Supplementary material for: TIAM-1/GEF can shape somatosensory dendrites independently of its GEF activity by regulating F-actin localization
Source: eLife. 2019 Jan 29;8:e38949. doi: 10.7554/eLife.38949 (PMC6370339; doi:10.7554/eLife.38949)
Supplement: Supplementary file 1 — In addition, Supplementary file 1 lists all transgenic strains created during the course of these studies. [file elife-38949-supp1.docx]

**Supplementary Material**

**To**

**TIAM-1/GEF can shape somatosensory dendrites independently of its GEF activity by regulating F-actin localization**

Leo T.H. Tang^1,3^, Carlos A. Díaz-Balzac^1,3^, Maisha Rahman^2^, Nelson J. Ramirez-Suarez^1^, Yehuda Salzberg^1§^, María I. Lázaro-Peña^1^, and Hannes E. Bülow^1,2^

^1^Department of Genetics and ^2^Dominick P. Purpura Department of Neuroscience

Albert Einstein College of Medicine

Bronx, New York, 10461, USA

^3^ contributed equally

* corresponding author: Telephone 718 430 3621, Fax 718 430 8778,

e-mail: [hannes.buelow@einstein.yu.edu](mailto:hannes.buelow@einstein.yu.edu)

^§^ present address : Weizmann Institute, Rehovot, 7610001, Israel

Running title: F-actin in dendrite patterning

7 Figures

7 Figure supplements

**Supplementary Information**

***Strain list***

***Fluorescent reporter strains***

PVD: *wdIs52* [*F49H12.4p*::GFP] II (kind gift of David Miller)

PVD: *wyIs378* [*ser-2p3::MYR-GFP + rab-3p::MYR-mCherry*] II (kind gift of Kang Shen)

PVD: *dzIs53* [*F49H12.4p*::mCherry]

Touch receptor neurons: *muIs32* [*mec-7p*::GFP] II

DMA-1::GFP in PVD: *qyIs369[ser-2p3::dma-1::GFP]* (kind gift of David Miller)

TIAM-1::GFP in PVD: *wyIs1139 [ser-2p3>tiam-1::GFP, Pmyo-2>mCherry]* (kind gift of Kang Shen)

***Strains used in this study:***

NC1687: *wdIs52 II*

EB2981: *tiam-1(tm1556) I; wdIs52 II*

EB1278: *tiam-1(dz184) I; wdIs52 II*

EB2566: *tiam-1(dz206) I; wdIs52 II*

EB2982: *tiam-1(ok772) I; wdIs52 II*

EB2798: *wdIs52 II; hpo-30(ok2047) V*

EB1269: *wdIs52 II; hpo-30(dz189) him-5(e1490) V*

EB1279: *wdIs52 II; hpo-30(dz178) V*

EB2984: *wdIs52 II; act-4(dz222) X*

EB2985*: wdIs52 II; act-4(gk279371) X*

EB2986: *wdIs52 II; act-4(*g*k279385) X*

EB2987: *wdIs52 II; act-4(gk473333) X*

EB2988: *wdIs52 II; act-4(gk785720) X*

EB2730: *lect-2(rz2) wdIs52 II*

EB1564: *dma-1(tm5159 )I; wdIs52 II*

EB1271: *wdIs52 II; mnr-1(dz175) V*

EB1899: *wdIs52 II; sax-7(nj48) IV*

EB1470: *kpc-1(gk8) I; wdIs52 II*

EB1649: *wdIs52 II; dzIs43*

EB2990: *tiam-1(tm1556) I; wdIs52 II; dzIs43*

EB2991: *wdIs52 II; hpo-30(ok2047) V; dzIs43*

EB2992: *wdIs52 II; act-4(dz222) X; dzIs43*

CF702: *muIs32 II*

EB2993: *muIs32 II; act-4(dz222) X*

EB2994: *tiam-1(tm1556) I; muIs32 II*

EB2995: *muIs32 II; hpo-30(ok2047) V*

EB2996: *tiam-1(tm1556) I; wdIs52 II; mnr-1(dz175) V*

EB2997: *tiam-1(tm1556) I; wdIs52 II; sax-7(nj48) IV*

EB2998: *tiam-1(tm1556) dma-1(tm5159) I; wdIs52 II*

EB2999: *tiam-1(tm1556) kpc-1(gk8) I; wdIs52 II*

EB3001: *tiam-1(tm1556) I; lect-2(rz2) wdIs52 II*

EB3002: *tiam-1(tm1556) I; wdIs52 II; hpo-30(ok2047) V*

EB3003: *tiam-1(tm1556) I; wdIs52 II; act-4(dz222) X*

EB3004*: wdIs52 II; sax-7(nj48) IV; hpo-30(ok2047) V*

EB3005*: dma-1(tm5159) I; wdIs52 II; hpo-30(ok2047) V*

EB2808: *dzIs53 ddIs290 II; him-5(ok1896) V*

EB3006: *dzIs53 ddIs290 II; hpo-30(ok2047) V*

EB3007: *dzIs53 ddIs290 II; act-4(dz222) X*

EB3171 *: tiam-1(tm1556) I; dzIs53 ddIs290 II*

EB2724*: lect-2(dz249)[lect-2::mNG^3xFlag]) II*

EB3008*: lect-2(dz249)[lect-2::mNG^3xFlag]) II; hpo-30(ok2047) V*

EB3009*: lect-2(dz249)[lect-2::mNG^3xFlag]) II; act-4(dz222) X*

EB3172: *tiam-1(tm1556) I; lect-2(dz249)[lect-2::mNG^3xFlag]) II*

EB3010*: wyEx4286; tiam-1(tm1556) I*

EB3011*: wyEx4286; hpo-30(ok2047) V*

EB3012*: wyEx4286; act-4(dz222) X*

EB3013*: tiam-1(dz264)[TIAM-1 T548F] I; wdIs52 II*

EB3014*: tiam-1(dz265)[TIAM-1 T548F] I; wdIs52 II*

EB2960: *dzEx1566; wdIs52 II*

EB2980: *dzEx1566; tiam-1(tm1556) I; wdIs52 II*

EB3017: *dzEx1566; wdIs52 II; act-4(dz222) X*

EB3058: *dzEx1566; dma-1(tm5159) I; wdIs52 II*

EB3167: *dzEx1566; wdIs52 II; hpo-30(ok2047) V*

EB3175: *dzEx1566; wdIs52 II; sax-7(nj48) IV*

EB2975: *dzEx1569; wdIs52 II*

EB3062: *dzEx1569; dma-1(tm5159) I; wdIs52 II*

EB3063: *dzEx1569; tiam-1(tm1556) I; wdIs52 II*

EB3168: *dzEx1569; wdIs52 II; hpo-30(ok2047) V*

EB3169: *dzEx1569; wdIs52 II; act-4(dz222) X*

EB3170: *dzEx1569; wdIs52 II; sax-7(nj48) IV; him-5(ok1869) V*

EB2871: *dzIs53 II; act-4(dz222) X*

EB2933: *dzEx1545; dzIs53 II; act-4(dz222) X*

EB2936: *dzEx1548; dzIs53 II; act-4(dz222) X*

EB2940: *dzEx1552; dzIs53 II; act-4(dz222) X*

EB2953: *tiam-1(dz206) I; wdIs52 II; act-4(dz222) X*

EB2693: *dma-1(dz244[dma-1::mNG^3xFlag]) I; dzIs53 II*

EB2945: *dzEx1555; wdIs52 II*

EB3015*: dzIs95*

EB3016*: dzIs95; wdIs52 II; act-4(dz222) X*

EB3176*: dma-1(tm5159) I; wdIs52 II; dzEx1571*

EB3183: *dma-1(dz266[ΔPDZ]) I; wdIs52 II*

TV22165: *wyIs1139*

EB3469: *dma-1(dz266) I; wyIs1139 II, dzEx1841*

EB3497: *qyIs369 X*

EB3426: *hpo-30(ok2047) V; qyIs369 X*

EB3448: *qyIs369 X; dzEx1837*

EB3495: *wyIs1130/dzIs53 II*

**List of plasmids used in transgenic experiments**

| **Plasmids for transgenesis** | **Source** |
| --- | --- |
| *ser-2p3s::dma-1* | The *dma-1* cDNA was first cloned into a plasmid with the full version of the *ser-2p3* including 5’ intronic regions located upstream of the start codon and used to drive expression in *C. elegans* plasmids. This plasmid (*ser2p3::5’intron::dma-1*) was used to subclone the *dma-1* cDNA sequence into *ser2p3s::5’intron::gfp* with Kpn*I* and Apa*I* to generate *ser2p3s::5’intron::dma-1*. A PCR fragment (2961bp) was obtained using DNA from *wyEx4286* (kind gift of Kang Shen) using the following primers: tgtggactggactttttggagac and ggttggctgtgatgtcgaaag. The resulting fragment was cloned into the *ser2p3s::5’intron::dma-1* plasmid with *Kpn2I* and *SalI* to generate *ser2p3s::dma-1*. For a description of the *ser-2p3s* promoter see Ramirez-Suarez *et al.*, 2019. |
| *F49H12.4p::dma-1ΔICD::mCherry* | A PCR fragment containing a C-terminal portion of DMA-1 lacking the intracellular domain was fused in frame to *mCherry* using a *dpy-7p::mCherry* plasmid as template with the following primers:  cttggatgcatctatttccttcgtgaggtgagcaagggcgaggag and  ggaattctagattacttgtacagctcgtccatgcc.  The resulting fragment was cloned into *ttx-3p::dma-1* using *NsiI* and *XbaI* and the *dma-1*ΔICD*::mCherry* fragment was further subcloned into a  *F49H12.4p::GFP* plasmid with *KpnI* and *XbaI* to generate  *F49H12.4p::dma-1*ΔICD::*mCherry*. |
| *ser-2p3s::dma-1ΔICD:: mCherry* | From the *F49H12.4p::dma-1ΔICD*::*mCherry* plasmid a fragment encoding the C-terminal part of *dma-1ΔICD*::*mCherry* and *unc-54* 3’UTR was subcloned into  *ser2p3s::dma-1* with *Kpn2I* and *ApaI*. |
| *ttx-3p::tiam-1* | The *tiam-1* cDNA was PCR amplified with *KpnI/XbaI* cloning sites attached from N2 cDNA and cloned into *ttx-3p::lect-2* using the following primers:  aaaaaaggtaccatgggctcacgcctctcatgttcttgc and aaaaaatctagactattttgaatttcgatgttccttag. The *tiam-1* cDNA sequence was confirmed by Sanger sequencing. |
| *ser-2p3::tiam-1* | The *tiam-1 cDNA* from *ttx-3p::tiam-1* was cloned into *ser-2p3::sax-7S* with *KpnI* and *ApaI*. |
| *rab-3p::tiam-1* | The *tiam-1 cDNA* from *ttx-3p::tiam-1* was cloned into *rab-3p::sax-7S* with  *KpnI* and  *ApaI* . |
| *dpy-7p::tiam-1* | The *tiam-1 cDNA* from *ttx-3p::tiam-1* was cloned into *dpy-7p::sax-7* with  *KpnI* and  *ApaI* . |
| *myo-3p::tiam-1* | The *tiam-1 cDNA* from *ttx-3p::tiam-1* was cloned into *myo-3p::sax-7* with  *KpnI* and  *ApaI* . |
| *ser-2p3::tiam-1(GEF-only)* | Site-directed Mutagenesis was performed with the Agilent Quickchange II XL Kit with the following primers: aaaaaggtaccatgggttgcctgttttcaaaagagcggcgagcaccgtctcaactcgacg and aaaaaatctagactattttgaatttcgatgttccttag on the *ttx-3p::tiam-1* and confirmed by Sanger sequencing. The *tiam-1(GEF-only)* was then subcloned into *ser-2p3::tiam-1* with *KpnI* and *ApaI*. |
| *ser-2p3::tiam-1(T548F)* | Site-directed Mutagenesis was performed with the Agilent Quickchange II XL Kit with the following primers: gctctgcaagaattgttggtctttgagaagaaatatgtcagcgatcttcgag and ctcgaagatcgctgacatatttcttctcaaagaccaacaattcttgcagagc on the *ttx-3p::tiam-1* and confirmed by sanger sequencing. The *tiam-1(T548F)* was then subcloned into *ser-2p3::tiam-1* with *KpnI* and *ApaI*. |
| *ser-2p3::tiam-1::mCherry* | A *tiam-1::mCherry* fragment was PCR amplified by PCR fusion and cloned into *ttx-3p::tiam-1* with *NsI* and *XbaI,* using the following primers: reaction 1 gaagttgatatcaacgaaatg and cctcctcgcccttgctcaccatttttgaatttcgatgttccttag, reaction 2 aggacccttggagggtacc and agttggtaatggtagcgacc, and fusion reaction gccaaatgatgcattaaatctaaaac and aaaaaatctagattacttgtacagctcgtcca. The *tiam-1-mcherry* fragment was digested with *NsI* and *XbaI* and cloned into *ttx-3p::tiam-1* and, further subcloned into  *ser-2p3::tiam-1* with *KpnI* and *ApaI*. |
| *ser-2p3::tiam-1(GEF-only)::mCherry* | A *tiam-1-mCherry* fragment was digested with *NsI* and *XbaI* from *ttx-3p::tiam-1* and cloned into *ttx-3p::tiam-1(GEF-only)*, creating *ttx-3p::tiam-1(GEF-only)::mCherry*. This was then subcloned into *ser-2p3::tiam-1 with KpnI* and *ApaI*. |
| *ser-2p3::act-4* | The *act-4* genomic DNA was amplified from N2 genomic DNA with primers atagaacattttcaggaggacccttggagggtaccatgtgtgacgacgaggttgccgctc and ttggaattctagatcagacaaaggcgggttccggattagaagcacttgcggtggacaatc, then inserted into *KpnI/BspEI* linearized *ser2p3::tiam-1* through Gibson Assembly. |
| *ser-2p3::act-1* | The *act-1* genomic DNA was amplified from N2 genomic preps with primers  atagaacattttcaggaggacccttggagggtaccatgtgtgacgacgaggttgcc  and tggaattctagatcagacaaaggcgggttccggattagaagcacttgcggtgaacgatg, then inserted into *KpnI/BspEI* linearized *ser2p3::tiam-1* through Gibson Assembly. |
| *myo-3p::act-4* | The *act-4* genomic DNA was amplified from *ser2p3::act-4* with primers atagaacattttcaggaggacccttggagg and tttcaggaggacccttggagg, then inserted into *KpnI/EcoRI* linearized *myo3p::tagBFP* through Gibson Assembly. |
| *act-4ap::NLS::mCherry* | The *act-4* promoter region (2.6 kb upstream) was amplified from N2 genomic DNA with primers cgctaacaacttggaaatgaaataagcttgcatgctcgaaaatcggcagccacatgc and aaacatacctttgggtcctttggccaatcccgggttttttgggaactgaaagcgggtgcc, then inserted into *SphI/XmaI* linearized *rab-3p::NLS::mCherry* through Gibson Assembly. |
| *ser-2p3::UtrCH::tagRFP* | *UtrCH* and *tagRFP* was amplified from *PGFP::UtrCH* and *unc-122p:stretavedin::tagRFP* with the following primer pairs: cgagctcaagcttcgaattccaacacgcgtaccatggccaagtatggagaac/ttggaattctagatcagacaaaggcgggttccggattagtctatggtgacttgctgagg and atagaacattttcaggaggacccttggagggtaccatggtgtctaagggcgaagagctg/acgcgtgttggaattcgaagcttgagctcgag, respectively. They were then ligated and inserted into *KpnI/BspEI* linearized *ser2p3::act-4* through Gibson Assembly. |
| *ser-2p3::tba-1::tagRFP* | *tba-1* and *tagRFP* were amplified from *ttx-3p::tba-1* and *unc-122p:streptavedin::tagRFP* with primers pairs cgagctcaagcttcgaattccaacacgcgtatgcgtgaggtcatctccatc/ttggaattctagatcagacaaaggcgggttccggattaatactcttctccttcctcctcg and atagaacattttcaggaggacccttggagggtaccatggtgtctaagggcgaagagctg/acgcgtgttggaattcgaagcttgagctcgag respectively. They were then ligated and inserted into *KpnI/BspEI* linearized *ser2p3::act-4* through Gibson Assembly. |
| *ser-2p3::hpo-30::tagBFP* | The *tagBFP* and *hpo-30* fragments were amplified from *myo-3p::tagBFP* and *ttx-3p::hpo-30*  with primer pairs ctcgagatggtcagcaagggagaggca/tggaattctagatcagacaaaggcgggttccggattaattaagcttgtgacccagtttgc atagaacattttcaggaggacccttggagg/aatcagctcttcgcccttagacaccatccatggcatactgctgtcatcgtcaatcactac. They were then ligated and inserted into *KpnI/BspEI* linearized *ser2p3::act-4* through Gibson Assembly. |
| *PPCDNA::dma-1::V5* | The *dma-1 cDNA* from *Pttx-3::dma-1* was cloned into *PPCDNA::sax-7::V5* with  *KpnI* and *XbaI*. The V5 tag was then introduced 5’ to the PDZ binding site through Quickchange site-directed mutagenesis with the following primer pairs:  caaaacctggatcatccaagcctatccctaaccc/gggttagggataggcttggatgatccaggttttg and  gggtttaaactcaatggtgatggtgatgatgctagatcccgaaatacgtagaatcgagaccgaggagagggttaggg/ccctaaccctctcctcggtctcgattctacgtatttcgggatctagcatcatcaccatcaccattgagtttaaaccc. |
| *PPCDNA::dma-1 (ΔPDZ)::V5* | A stop codon was introduced to *PPCDNA::dma-1::V5* at the start of the PDZ binding site (YFGI motif) through quickchange site-directed mutagenesis with primers ggtctcgattctacgtagtttggcattcgtaccgg and ggtctcgattctacgtagtttggcattcgtaccgg. |
| *PPCDNA::dma-1 (ΔICD)::V5* | A truncation between R531 to C-terminus was introduced in *PPCDNA::dma-1::V5* through Quickchange site-directed mutagenesis with primers ccgcgggccctcaaggaaatagatgcatccaaga and tcttggatgcatctatttccttgagggcccgcgg. |
| *PPCDNA::hpo-30::HA* | The Stop codon of *Pttx-3::hpo-30* was mutagenized with site-directed Mutagenesis performed with the Agilent Quickchange II XL Kit with the following primers: ctcagttggaattctagacacatactgctgtcatcg and gatgacagcagtatgtgtctagaattccaactgag. The *hpo-30 cDNA* without stop codon from *Pttx-3::hpo-30* was cloned into *PPCDNA::sax-7::HA* with  *KpnI* and *XbaI*. |
| *PPCDNA::tiam-1::HA* | The *tiam-1 cDNA* from *Pttx-3::tiam-1* was cloned into *PPCDNA::sax-7::HA* with  *KpnI* and *XbaI*. The HA tag was then made in-frame with site-directed Mutagenesis performed with the Agilent Quickchange II XL Kit with the following primers: ctaaggaacatcgaaattcaaaatatctagagggcccg and cgggccctctagatattttgaatttcgatgttccttag. |
| *PPCDNA::tiam-1 ΔEVH1::HA* | *tiam-1* domain mutant were amplified from their respective *Pttx-3::[tiam-1 mutant]::mCherry* with primers  agggagacccaagctggctagttaagcttgggtaccatgggctcacgcct  and ggcttaccttcgaaccgcgggccctctagatattttgaatttcgatgttccttagagact, then inserted into *KpnI/XbaI* linearized *PPCDNA*::*tiam-1:HA* through Gibson Assembly |
| *PPCDNA::tiam-1ΔPDZ::HA* |  |
| *PPCDNA::tiam-1ΔDH1::HA* |  |
| *PPCDNA::tiam-1ΔPH1::HA* |  |
| *PPCDNA::act-4::V5* | The *act-4* cDNA was amplified from *yk1520h11* with primers  agggagacccaagctggctagttaagcttggtaccatgtgtgacgacgaggttgccgctc  and ggataggcttaccttcgaaccgcgggccctctagattagaagcacttgcggtggacaatc and ligated into *KpnI/Xba1* linearized *PPCDNA::dma-1::V5*  through Gibson Assembly. The V5 tag was then inserted in-frame with site-directed mutagenesis with primers  cgggccctctagattgaagcacttgcggtg and caccgcaagtgcttcaatctagagggcccg |
| *PPCDNA::act-4 G151E::V5* | The G151E mutation was introduced to *PPCDNA::act-4::V5* through Quickchange site-directed mutagenesis using primers cgggccctctagattgaagcacttgcggtg and caccgcaagtgcttcaatctagagggcccg. |
| *PPCDNA::act-4::FLAG* | The *act-4* cDNA were and isolated from *PPCDNA::act-4::V5* with *KpnI/XbaI* and ligated into *KpnI/Xba1* linearized *PPCDNA::mig-10::FLAG* |

**List of transgenic strains with genotypes and strain names**

| **Strain name** | **Constructs** | **Genotype** | ***Line^a^*** |
| --- | --- | --- | --- |
| EB3018 | *ser-2p3::tiam-1* & *myo-3p::mCherry* | *dzEx1572; tiam-1(tm1556) I; wdIs52 II* | *1* |
| EB3019 | *ser-2p3::tiam-1* and *myo-3p::mCherry* | *dzEx1572; tiam-1(tm1556) I; wdIs52 II* | *2* |
| EB3020 | *ser-2p3::tiam-1* and *myo-3p::mCherry* | *dzEx1573; tiam-1(tm1556) I; wdIs52 II* | *3* |
| EB3021 | *ser-2p3::tiam-1* and *myo-3p::mCherry* | *dzEx1574; tiam-1(tm1556) I; wdIs52 II* | *4* |
| EB3022 | *rab-3p::tiam-1* and *myo-3p::mCherry* | *dzEx1575; tiam-1(tm1556) I; wdIs52 II* | *1* |
| EB3023 | *rab-3p::tiam-1* and *myo-3p::mCherry* | *dzEx1576; tiam-1(tm1556) I; wdIs52 II* | *2* |
| EB3024 | *rab-3p::tiam-1* and *myo-3p::mCherry* | *dzEx1577; tiam-1(tm1556) I; wdIs52 II* | *3* |
| EB3025 | *myo-3p::tiam-1* and *myo-3::mCherry* | *dzEx1578; tiam-1(tm1556) I; wdIs52 II* | *1* |
| EB3026 | *myo-3p::tiam-1* and *myo-3p::mCherry* | *dzEx1579; tiam-1(tm1556) I; wdIs52 II* | *2* |
| EB3027 | *myo-3p::tiam-1* and *myo-3p::mCherry* | *dzEx1580; tiam-1(tm1556) I; wdIs52 II* | *3* |
| EB3028 | *myo-3p::tiam-1* and *myo-3p::mCherry* | *dzEx1581; tiam-1(tm1556) I; wdIs52 II* | *4* |
| EB3029 | *myo-3p::tiam-1* and *myo-3p::mCherry* | *dzEx1582; tiam-1(tm1556) I; wdIs52 II* | *5* |
| EB3030 | *dpy-7p::tiam-1* and *myo-3p::mCherry* | *dzEx1583; tiam-1(tm1556) I; wdIs52 II* | *1* |
| EB3031 | *dpy-7p::tiam-1* and *myo-3p::mCherry* | *dzEx1584; tiam-1(tm1556) I; wdIs52 II* | *2* |
| EB3032 | *dpy-7p::tiam-1* and *myo-3p::mCherry* | *dzEx1585; tiam-1(tm1556) I; wdIs52 II* | *3* |
| EB3033 | *dpy-7p::tiam-1* and *myo-3p::mCherry* | *dzEx1586; tiam-1(tm1556) I; wdIs52 II* | *4* |
| EB3034 | *dpy-7p::tiam-1* and *myo-3p::mCherry* | *dzEx1587; tiam-1(tm1556) I; wdIs52 II* | *5* |
| EB3035 | *dpy-7p::tiam-1* and *myo-3p::mCherry* | *dzEx1588; tiam-1(tm1556) I; wdIs52 II* | *6* |
| EB3036 | *ser-2p3::tiam-1(T to F)* & *myo-p3::mCherry* | *dzEx1589; tiam-1(tm1556) I; wdIs52 II* | *1* |
| EB3037 | *ser-2p3::tiam-1(T to F* & *myo-p3::mCherry* | *dzEx1590; tiam-1(tm1556) I; wdIs52 II* | *2* |
| EB3038 | *ser-2p3::tiam-1(T to F)* & *myo-p3::mCherry* | *dzEx1591; tiam-1(tm1556) I; wdIs52 II* | *3* |
| EB3039 | *ser-2p3::tiam-1(GEF-only)* & *myo-p3::mCherry* | *dzEx1592; tiam-1(tm1556) I; wdIs52 II* | *1* |
| EB3040 | *ser-2p3::tiam-1(GEF-only)* & *myo-p3::mCherry* | *dzEx1593; tiam-1(tm1556) I; wdIs52 II* | *2* |
| EB3041 | *ser-2p3::tiam-1(GEF-only)* & *myo-p3::mCherry* | *dzEx1594; tiam-1(tm1556) I; wdIs52 II* | *3* |
| EB3042 | *ser-2p3::tiam-1(GEF-only)* & *myo-p3::mCherry* | *dzEx1595; tiam-1(tm1556) I; wdIs52 II* | *4* |
| EB3043 | *ser-2p3::tiam-1(GEF-only)* & *myo-p3::mCherry* | *dzEx1596; tiam-1(tm1556) I; wdIs52 II* | *5* |
| EB3044 | *ser-2p3::tiam-1::mCherry* & *myo-p3::mCherry* | *dzEx1597; tiam-1(tm1556) I; wdIs52 II* | *1* |
| EB3045 | *ser-2p3::tiam-1::mCherry* & *myo-p3::mCherry* | *dzEx1598; tiam-1(tm1556) I; wdIs52 II* | *2* |
| EB3046 | *ser-2p3::tiam-1::mCherry* & *myo-p3::mCherry* | *dzEx1599; tiam-1(tm1556) I; wdIs52 II* | *3* |
| EB3047 | *ser-2p3::tiam-1::mCherry* & *myo-p3::mCherry* | *dzEx1600; tiam-1(tm1556) I; wdIs52 II* | *4* |
| EB3048 | *ser-2p3::tiam-1(GEF-only)::mCherry* and  *myo-3p::mCherry* | *dzEx1601; tiam-1(tm1556) I; wdIs52 II* | *1* |
| EB3049 | *ser-2p3::tiam-1(GEF-only)::mCherry* and  *myo-3p::mCherry* | *dzEx1602; tiam-1(tm1556) I; wdIs52 II* | *2* |
| EB3050 | *ser-2p3::tiam-1(GEF-only)::mCherry* and  *myo-3p::mCherry* | *dzEx1603; tiam-1(tm1556) I; wdIs52 II* | *3* |
| EB3051 | *ser-2p3::tiam-1(GEF-only)::mCherry* and  *myo-3p::mCherry* | *dzEx1604; tiam-1(tm1556) I; wdIs52 II* | *4* |
| EB3052 | *ser-2p3::tiam-1(GEF-only)::mCherry* and  *myo-3p::mCherry* | *dzEx1605; tiam-1(tm1556) I; wdIs52 II* | *5* |
| EB3053 | *ser-2p3::tiam-1(GEF-only)::mCherry* and  *myo-3p::mCherry* | *dzEx1606; tiam-1(tm1556) I; wdIs52 II* | *6* |
| EB3054 | *ser-2p3::tiam-1(GEF-only)::mCherry* and  *myo-3p::mCherry* | *dzEx1607; tiam-1(tm1556) I; wdIs52 II* | *7* |
| EB3055 | *ser-2p3::tiam-1(GEF-only)::mCherry* and  *myo-3p::mCherry* | *dzEx1608; tiam-1(tm1556) I; wdIs52 II* | *8* |
| EB2933 | *myo-3p::act-4 and elt-2p::gfp* | *dzEx1545; dzIs53 II; act-4(dz222) X* | *1* |
| EB2934 | *myo-3p::act-4 and elt-2p::gfp* | *dzEx1546; dzIs53 II; act-4(dz222) X* | *2* |
| EB2935 | *myo-3p::act-4 and elt-2p::gfp* | *dzEx1547; dzIs53 II; act-4(dz222) X* | *3* |
| EB2936 | *ser-2p3::act-4 and elt-2p::gfp* | *dzEx1548; dzIs53 II; act-4(dz222) X* | *1* |
| EB2937 | *ser-2p3::act-4 and elt-2p::gfp* | *dzEx1549; dzIs53 II; act-4(dz222) X* | *2* |
| EB2938 | *ser-2p3::act-4 and elt-2p::gfp* | *dzEx1550; dzIs53 II; act-4(dz222) X* | *3* |
| EB2939 | *ser-2p3::act-4 and elt-2p::gfp* | *dzEx1551; dzIs53 II; act-4(dz222) X* | *4* |
| EB2940 | *ser-2p3::act-1 and elt-2p::gfp* | *dzEx1552; dzIs53 II; act-4(dz222) X* | *1* |
| EB2941 | *ser-2p3::act-1 and elt-2p::gfp* | *dzEx1553; dzIs53 II; act-4(dz222) X* | *2* |
| EB2942 | *ser-2p3::act-1 and elt-2p::gfp* | *dzEx1554; dzIs53 II; act-4(dz222) X* | *3* |
| EB2945 | *act-4p1::tagRFP and myo-p2::mNG* | *dzEx1555; wdIs52 II* | *1* |
| EB2946 | *act-4p1::tagRFP and myo-2p::mNG* | *dzEx1556; wdIs52 II* | *2* |
| EB2947 | *act-4p1::tagRFP and myo-2p::mNG* | *dzEx1557; wdIs52 II* | *3* |
| EB2958 | *ser-2p3::tagRFP::UtrCH & myo-3p::tagBFP* | *dzEx1564; wdIs52 II* | *1* |
| EB2959 | *ser-2p3::tagRFP::UtrCH & myo-3p::tagBFP* | *dzEx1565; wdIs52 II* | *2* |
| EB2960 | *ser-2p3::tagRFP::UtrCH & myo-3p::tagBFP* | *dzEx1566; wdIs52 II* | *3* |
| EB2973 | *ser-2p3::tagRFP::tba-1 & myo-3p::tagBFP* | *dzEx1567; wdIs52 II* | *1* |
| EB2974 | *ser-2p3::tagRFP::tba-1 & myo-3p::tagBFP* | *dzEx1568; wdIs52 II* | *2* |
| EB2975 | *ser-2p3::tagRFP::tba-1 & myo-3p::tagBFP* | *dzEx1569; wdIs52 II* | *3* |
| EB2976 | *ser-2p3::tagRFP::tba-1 & myo-3p::tagBFP* | *dzEx1570; wdIs52 II* | *4* |
| EB3447 | *ser-2p3::hpo-30::tagBFP & myo-2p::mCherry* | *qyIs369 X; dzEx1836* | *1* |
| EB3448 | *ser-2p3::hpo-30::tagBFP & myo-2p::mCherry* | *qyIs369 X; dzEx1837* | *2* |
| EB3177 | *ser-2p3s::dma-1 & myo-3p::tagRFP* | *dma-1(tm5159) I; wdIs52; dzEx1694* | *1* |
| EB3178 | *ser-2p3s::dma-1 & myo-3p::tagRFP* | *dma-1(tm5159) I; wdIs52; dzEx1695* | *2* |
| EB3179 | *ser-2p3s::dma-1 & myo-3p::tagRFP* | *dma-1(tm5159) I; wdIs52; dzEx1696* | *3* |
| EB3180 | *ser-2p3s::dma-1*ΔICD::*mCherry and*  *myo-2p::mCherry* | *dma-1(tm5159) I; wdIs52; dzEx1697* | *1* |
| EB3181 | *ser-2p3s::dma-1*ΔICD::*mCherry and*  *myo-2p::mCherry* | *dma-1(tm5159) I; wdIs52; dzEx1698* | *2* |
| EB3469 | *F49H12.4p::mCherry, myo-3p::tagBFP* | *dma-1(dz266) I; wyIs1139 II, dzEx1841* |  |

^a^ line refers to the respective numbering of extrachromosomal transgenic lines used.

**List of antibodies used in Co-immunoprecipitation experiments**

| **Antibody** | **Type** | **Source** | **Catalogue #** |
| --- | --- | --- | --- |
| Anti-HA | Rat monoclonal primary | Sigma | ROAHAHA |
| Anti-Flag | Mouse monoclonal primary | Sigma | F3165 |
| Anti-V5 | Mouse monoclonal primary | Thermo-Fisher | R960-25 |
| Anti-Rat HRP | Secondary | Thermo-Fisher | 31470 |
| Anti-mouse HRP | Secondary | Millipore | AP308P |
